# Norepinephrine modulates IL-1β-induced catabolic response of human chondrocytes

**DOI:** 10.1186/s12891-021-04598-7

**Published:** 2021-08-23

**Authors:** Hyun Sook Hwang, Mi Hyun Lee, Dong Jin Go, Hyun Ah Kim

**Affiliations:** 1 Division of Rheumatology, Department of Internal Medicine, Hallym University Sacred Heart Hospital, 896, Pyungchon, Anyang, Kyunggi 14068 Korea; 2 Institute for Skeletal Aging, Hallym University, Chunchon, Gangwon 24251 Korea; 3 Division of Rheumatology, Department of Internal Medicine, Hallym University Kangnam Sacred Heart Hospital, Seoul, 07442 Korea

**Keywords:** Beta-adrenergic receptor, Cartilage, Interleukin 1-beta, Norepinephrine, Osteoarthritis, Sympathetic nerve system, β-blocker, Glycosaminoglycan, Chondrocyte

## Abstract

**Background:**

The influence of the sympathetic nervous system (SNS) on metabolism of bone and cartilage expressing β-adrenergic receptors (AR) was suggested. Here, we investigated whether the SNS functions as a modulator of cartilage metabolism induced by interleukin-1beta (IL-1β).

**Methods:**

Human articular chondrocytes and articular cartilage were collected from patients with osteoarthritis (OA). Chondrocyte monolayer and cartilage explant culture were stimulated with IL-1β. The activity of β-ARs was modulated by an agonist, norepinephrine (NE), and antagonists, including propranolol, atenolol, nebivolol, and nadolol.

**Results:**

The levels of β_1_-, β_2_-, and β_3_-AR in OA cartilage and IL-1β-treated chondrocytes were lower than normal cartilage and untreated cells. Treatment of chondrocytes with IL-1β and β-blockers, including propranolol, atenolol, nebivolol, and nadolol, for 6 h significantly upregulated IL-1β-induced expression of MMP-1, -3, and − 13, compared to chondrocytes treated with IL-1β alone, indicating that antagonism of β-AR confers catabolic signals. On the other hand, NE antagonized IL-1β-induced catabolic response. In addition, NE significantly inhibited IL-1β-induced release of glycosaminoglycan (GAG) from cartilage explant culture. In addition, β-AR activity significantly affected IL-1β-stimulated phosphorylation of JNK and ERK. These results indicate that β-AR signal is associated with cartilage metabolism.

**Conclusions:**

Our findings showed that β-ARs is a regulator of cartilage catabolism induced with IL-1β.

**Supplementary Information:**

The online version contains supplementary material available at 10.1186/s12891-021-04598-7.

## Background

Osteoarthritis (OA) is a prevalent degenerative joint disease, which shows features such as cartilage loss, synovitis, and accompanying joint pain [1]. Synthesis of extracellular matrix (ECM) in chondrocytes is maintained by balance of anabolic factors and catabolic factors, such as matrix metalloproteinases (MMPs), and are also influenced by a variety of molecules, including pro-inflammatory mediators and cytokines [1, 2].

Stimuli from inflammation and mechanical stress are detected by sensory nerve fibers and is transmitted to the central nervous system, which subsequently activates the sympathetic nervous system (SNS). A series of processes, including release of neurotransmitter such as norepinephrine (NE), lymphocyte recruitment, and increase of blood and lymph flow, are induced at the site of inflammation [3]. In particular, the sympathetic nerve fibers expressing α- and β-adrenergic receptors (AR) are found in various tissues, including synovium and bone. Previous studies reported results linking the SNS with the skeletal system. The endogenous catecholamine NE was detected at high concentration in synovial fluid from patients with joint trauma [4]. NE regulates bone development and chondrocyte metabolism via β-AR [5]. NE has also been shown to regulate apoptosis and proliferation of several cell types including chondrocytes via β-AR [6–8]. Of note, NE exhibits a distinct impact depending on the receptor it signals through such that inflammatory response of OA chondrocyte signals via β-AR while cell cycle and apoptosis signals via α-AR [9].

Furthermore, the influence of AR signaling on skeletal tissue homeostasis has been reported in animal and human studies. Intracerebroventricular administration of leptin reduced bone formation and bone mass in mice, with leptin acting on the hypothalamus to increase sympathetic outflow, leading to the activation of β-ARs on osteoblasts and resulting in decreases in osteoblast proliferation, differentiation, and bone formation [10, 11]. Treatment with the nonselective β-blocker propranolol increased bone mass in ovariectomized as well as ovary-intact female mice [11]. In addition, a study of postmenopausal women showed that patients treated with β1-AR–selective blockers had decrease of bone resorption marker and increase of bone mineral density than did nonusers [12]. However, it is little known whether the SNS signaling affects cartilage metabolism in chondrocytes or OA patients.

In this study, we investigated whether the regulation of β-AR activity by NE and its antagonists has an influence on interleukin-1β (IL-1β)-induced catabolic and anabolic responses in articular chondrocytes.

## Methods

### Materials

Recombinant human IL-1β was purchased from R&D Systems (Minneapolis, MN, USA). Propranolol (P), atenolol (A), nebivolol (B), and nadolol (N) were obtained from Sigma-Aldrich (St. Louis, MO, USA). Antibodies against p-IκBα, p-p38/p38, p-ERK/ERK, and p-JNK/JNK were purchased from Cell Signaling Technology (Danvers, MA, USA). Horseradish peroxidase (HRP)-conjugated secondary antibodies were obtained from Santa Cruz Biotechnology (Santa Cruz, CA, USA). Primers used in qRT-PCR were obtained from Cosmogenetech (Seoul, Korea).

### Cartilage collection and chondrocyte isolation from cartilage

OA cartilage samples were obtained from the knee joints of OA patients [n = 8, 74.25 ± 4.68 years] at the time of total knee replacement surgery. Patient diagnoses were determined using the criteria set forth by the American College of Rheumatology. Normal cartilage samples were obtained from the femoral head of patients [n = 6, 63.17 ± 12.81 years] with femoral neck fractures and no known history of OA or RA. Sample was obtained only from grossly normal-looking cartilage. The collection and use of human tissue samples was reviewed and approved by the Institutional Review Board of Hallym University Sacred Heart Hospital, Anyang, Korea (approval number 2018-05-040). All patients provided written informed consent. All methods were performed in accordance with the relevant guidelines and regulations of Hallym University and were approved by its ethical committee. Primary chondrocytes were isolated from articular cartilage as previously described [13]. In brief, articular cartilage tissues from a relatively lesion-free area were cut into small pieces and were serially digested with a protease from *Streptomyces griseus*, collagenase from *Clostridium histolysticum*, and hyaluronidase from bovine testes (Sigma-Aldrich). The digested cartilage suspension was passed through the cell strainer (20 μm) and the obtained chondrocytes were seeded at a density of 6 ~ 9 × 10^5^cells/ml in Dulbecco’s modified Eagle’s medium (DMEM) supplemented with 10 % of fetal bovine serum (FBS) and 1 % of penicillin/streptomycin at 37 °C in 5 % CO_2_ and 95 % air. First-passage cultured chondrocytes were used for all experiments within 1 week after seeding.

### Quantitative real-time reverse transcription polymerase chain reaction (qRT-PCR)

To examine whether the SNS affects IL-1β-induced cartilage catabolism, chondrocytes were pre-treated with β-blockers (0.1, 1, and 10 ng/ml) or NE (0.1, 1, and 10 ng/ml) for 2 h followed by incubation with IL-1β (1 ng/ml) for 6, 24 or 48 h. Total RNA was extracted from chondrocytes using TRIzol reagent as previously described [13]. Briefly, cDNA was synthesized from 2 µg of total RNA using Moloney murine leukemia virus reverse transcriptase (Promega, Madison, WI, USA). qRT-PCR was performed using a StepOnePlus real-time PCR system with the primers in Table S1. Glyceraldehyde 3-phosphate dehydrogenase (GAPDH), commonly used housekeeping gene, was used as an internal control because its mRNA expression in primary chondrocytes was unaffected in response to a variety of stimuli, including IL-1β.

### Immunohistochemical analysis

Normal and OA cartilage tissues were fixed in 4 % paraformaldehyde (PFA) for 48 h and embedded in paraffin and sectioned at a thickness of 5 μm using a microtome. For deparaffinization, the sections were sequentially placed in the following solutions; xylene, 100 %, 95 %, 90 %, 70 % ethanol, distilled water (DW), and phosphate buffered saline (PBS) for 3 min. The section were treated with hyaluronidase (2 mg/ml) at 37 °C for 30 min and were boiled in antigen retrieval solution for 5 min for antigen retrieval. The sections were blocked in blocking buffer (0.2 % BSA) for 30 min and incubated with primary antibodies against β_1_-, β_2_-, and β_3_-AR (1: 200 dilution) at 4 °C overnight, followed by incubation with biotinylated secondary antibody for 1 h at room temperature. Slides were treated with Vectastain ABC reagent (Vector Laboratories, Burlingame, CA, USA), visualized 3,3′-diaminobenzidine for 8 min, and counterstained with methyl green.

### Western blot analysis

To investigate the effect of β-blockers or NE on IL-1β-induced activation of MAPK and NF-κB, chondrocytes were pre-treated with β-blockers (1 ng/ml) or NE (1 ng/ml) for 2 h followed by incubation with IL-1β (1 ng/ml) for 15, 30, and 60 min. To examine the effect of β-blockers or NE on expression of catabolic and anabolic factor proteins, chondrocytes were treated with β-blockers (1 ng/ml) or NE (1 ng/ml) for 48 h with or without IL-1β (1 ng/ml). Chondrocytes was lysed with RIPA lysis buffer (Biosesang, Kyunggi, South Korea) and protein concentrations were determined using bicinchoninic acid protein assay (Thermo Fisher Scientific, Rockford, IL, USA). Equal amounts of proteins were separated by 10 % sodium dodecyl sulfate-polyacrylamide gel electrophoresis and blotted to a polyvinylidene difluoride membrane (Bio-Rad Laboratories, Hercules, CA, USA). The membrane was blocked with 5 % (w/v) nonfat milk in TBST and incubated with primary and secondary antibodies (1:1000 dilution). The antibody complex was detected using an enhanced chemiluminescence detection kit (Santa Cruz Biotechnology). The band intensities in all blots were measured by setting a threshold using densitometric program Image J software version 1.51 (National Institutes of Health, Bethesda, MD, USA, https://imagej.nih.gov/ij/download.html). The relative band density of target protein was calculated as dividing the intensity of each phosphate band by the intensity of total form, but by the intensity of β-actin band in case of p-IκBα.

### Enzyme-linked immunosorbent assay (ELISA)

The MMP-13 level in the medium was measured by ELISA using a pro-MMP-13 immunoassay kit according to the manufacturer’s instructions (R&D Systems). Briefly, 50 µl of standard solution and culture medium was added to each well and human Pro-MMP-13 conjugate (200 µl) was added to each well for 2 h at room temperature on shaker. Substrate solution (200 µl) was added to each well for 30 min at room temperature and the reaction was stopped by addition of Stop solution (50 µl). Optical density was measured at 450 nm using a Thermo Scientific Multiskan GO Microplate Spectrophotometer (Thermo Fisher Scientific, Vantaa, Finland). The concentration of MMP-13 in each sample was calculated from the standard curve.

### Explant culture

For explant culture, small pieces of articular cartilage tissue (40 mg) from a lesion-free area of cartilage were incubated in DMEM containing either PBS, IL-1β (1 ng/ml), IL-1β + NE (1 ng/ml), or IL-1β + β-blockers (1 ng/ml) for 1, 4, and 7 days. IL-1β-untreated cartilage was used as a control. The media was changed every 2 days during the culture period. Media were collected at desired time points.

### Safranin O staining

For measurement of proteoglycan content in the cartilage, Safranin O staining was performed as previously described. Briefly, paraffin-embedded cartilage sections were deparaffinized in xylene, hydrated through gradient 100 %, 95 %, 90, and 70 % ethyl alcohol, and stained in hematoxylin solution for 1 min. Then, the slides were counterstained with 0.05 % Fast green solution for 5 min, 1 % acetic acid solution, and 0.1 % Safranin O staining for 5 min.

## Measurement of glycosaminoglycan (GAG) released in culture medium

GAG content in sample was measured using Blyscan Sulfated Glycosaminoglycan kit (Biocolor Ltd., Antrim, UK) according to the manufacturer’s instructions. Briefly, Blyscan dye reagent (1.0 ml) was added to the same volume of medium from explant culture for 1, 4, and 7 days and glycosaminoglycan standard solution for a calibration curve followed by mixing by inverting and gentle shaking for 30 min. A sulfated GAG dye complex formed in each reaction tube was obtained by centrifugation at 12,000 rpm for 10 min. The pellets were dissolved in dissociation reagent (0.5 ml) and optical density was measured at 656 nm. GAG contents were calculated from the standard curve.

### Statistical analysis

Normality test of all data was performed using the Shapiro-Wilk test. Datasets that did not pass the normality test were subjected to Mann-Whitney test between two groups and Kruskal-Wallis test with post hoc Dunn’s test for multiple comparison. Data are expressed as the mean ± standard deviation (SD). All analyses were performed using GraphPad Prism 6.07 (GraphPad Software, San Diego, CA, USA, https://www.graphpad.com/scientific-software/prism). A value of *P* < 0.05 was considered statistically significant.

## Results

### The level of beta-adrenergic receptors (β-AR) is significantly decreased in OA cartilage

**Fig. 1:**
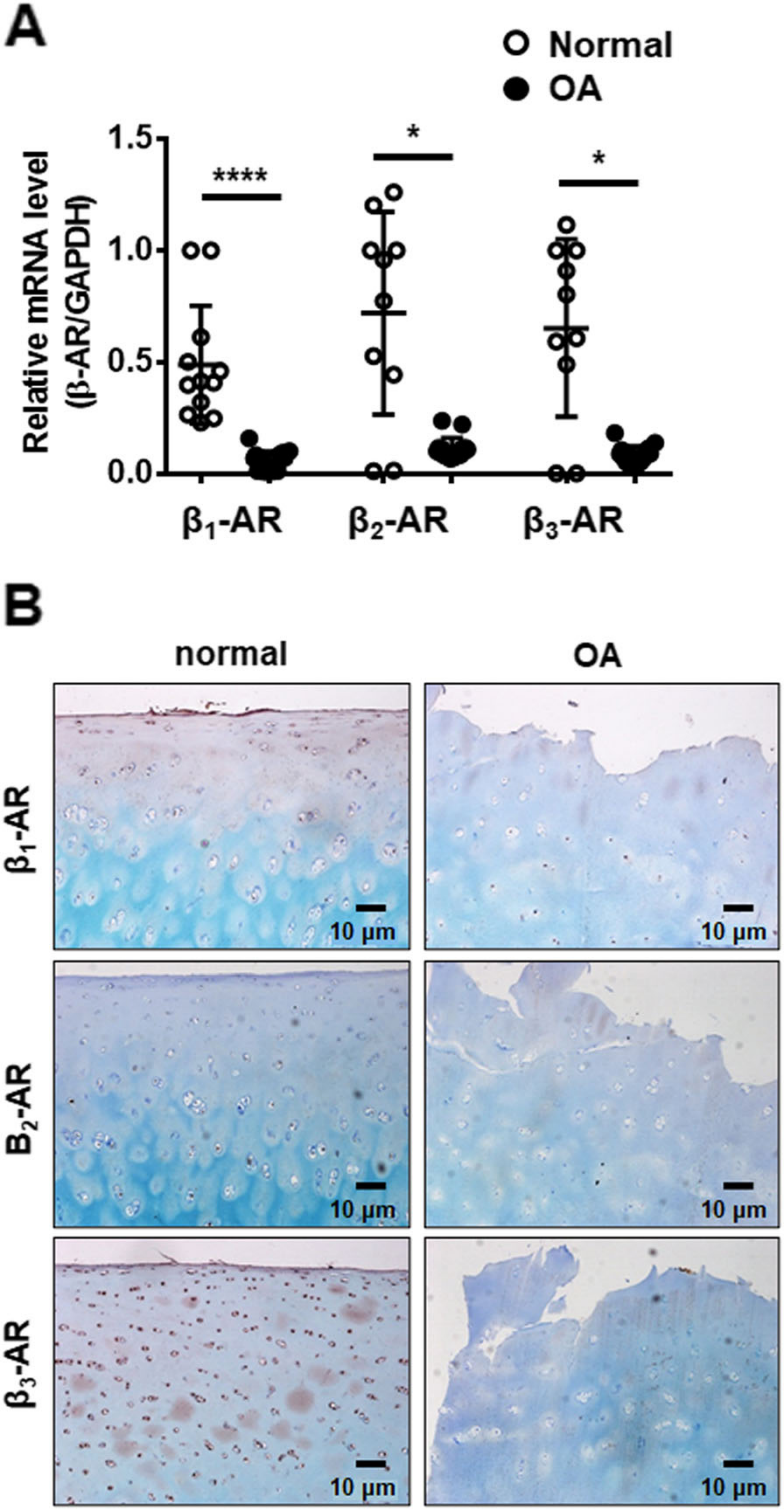
The levels of beta-adrenergic receptors are decreased in OA cartilage. (**A**) The relative expression of β_1_-, β_2_-, and β_3_-adrenergic receptor (AR) in normal and OA cartilage. Data represent the mean ± standard deviation (SD) for duplicate data from different donors. Normal cartilage (*n* = 6); OA cartilage (*n* = 8). *P < 0.05 and ****P < 0.0001 vs. normal cartilage tissue by Mann-Whitney test. (**B**) Decreased expression of β_1_-, β_2_-, and β_3_-AR proteins in OA cartilage. The levels of β_1_-, β_2_-, and β_3_-AR protein were measured by immunohistochemistry (IHC) using antibodies against β_1_-, β_2_-, and β_3_- AR. Scale bars = 10 μm

To investigate whether the level of subtypes of β-AR was different in normal and OA cartilage, mRNA levels of β-ARs were measured in normal and OA cartilages. The mRNA expression level of β_1_, β_2_, and β_3_-AR in normal cartilages varied depending on the donor, however, it was significantly lower in OA cartilage (Fig. 1A). IHC analysis also showed that protein expression of β_1_, β_2_, and β_3_-AR in OA cartilages was decreased compared to normal cartilage (Fig. 1B). These results demonstrate that the expression of β-ARs is downregulated in OA.

### The level of β-ARs is reduced in chondrocytes in response to IL-1β

**Fig. 2:**
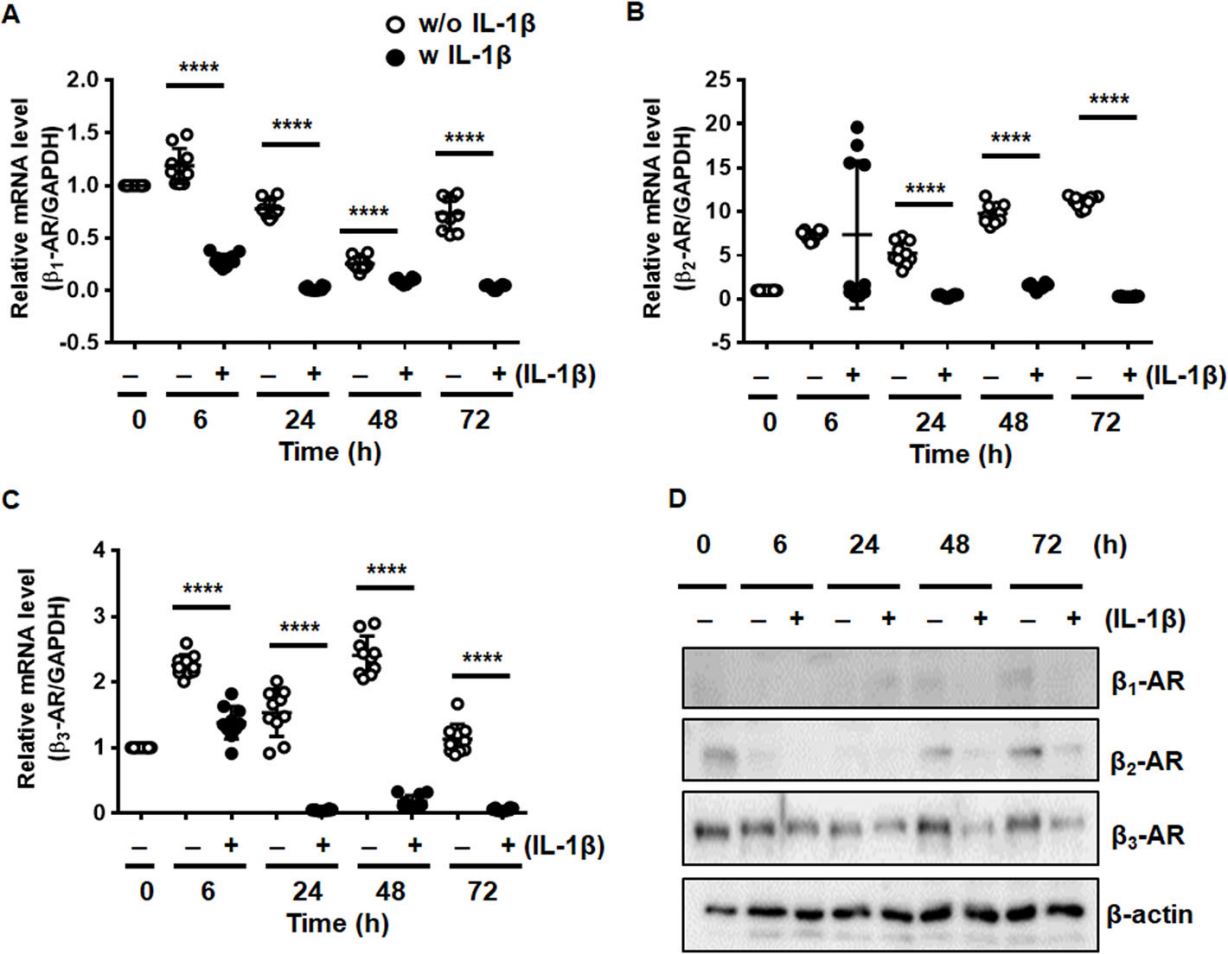
β-ARs were downregulated in response to IL-1β. (**A**)~(**C**) The mRNA levels of β_1_-, β_2_-, and β_3_-AR in IL-1β-treated chondrocytes. Chondrocytes were stimulated with IL-1β (1 ng/ml) for 6, 24, 48, and 72 h. mRNA levels of β-ARs were quantified using Quanti Fast SYBR Green-based real-time PCR (RT-PCR). The expression ratios of β-adrenergic receptors relative to glyceraldehyde 3-phosphate dehydrogenase (GAPDH), the internal control, are shown. Data represent the mean ± standard deviation (SD) for duplicate data from five different donors. ****P < 0.0001 vs. untreated chondrocytes by Mann-Whitney test. (**D**) Reduction of β_1_-, β_2_-, and β_3_-AR proteins in IL-1β-treated chondrocytes. Human chondrocytes were treated with IL-1β (1 ng/ml) for 6, 24, 48, and 72 h. Protein levels of β-ARs were measured using western blot analysis. β-actin was used as a loading control

To examine the effect of IL-1β on expression of β-ARs, chondrocytes were exposed to IL-1β (1 ng/ml) for 72 h. IL-1β treatment led to significantly decreased mRNA and protein expression of three β-AR subtypes, which lasted for 72 h in cultured chondrocytes (Fig. 2A-D). These results demonstrate that the expression of β-ARs is significantly reduced in chondrocytes in response to IL-1β.

To determine whether the activity of β-AR affects the expression of β-ARs in chondrocytes, the effect of β-adrenergic blockers and NE on the mRNA level of β-ARs was measured. Treatment with β-adrenergic blockers alone reduced the expression of β_1_, β_2_, and β_3_-AR compared with untreated control cells (Fig. S1A). On the contrary, NE highly increased (Fig. S1A). In addition, β-blockers had no influence on IL-1β-induced suppression of β-ARs but NE reversed it (Fig. S1B). The results demonstrate that activation of β-ARs by NE positively affects expression of β-ARs regardless of inflammatory condition.

### β-adrenergic blockers and NE modulate IL-1β-induced expression of catabolic and anabolic factors

**Fig. 3:**
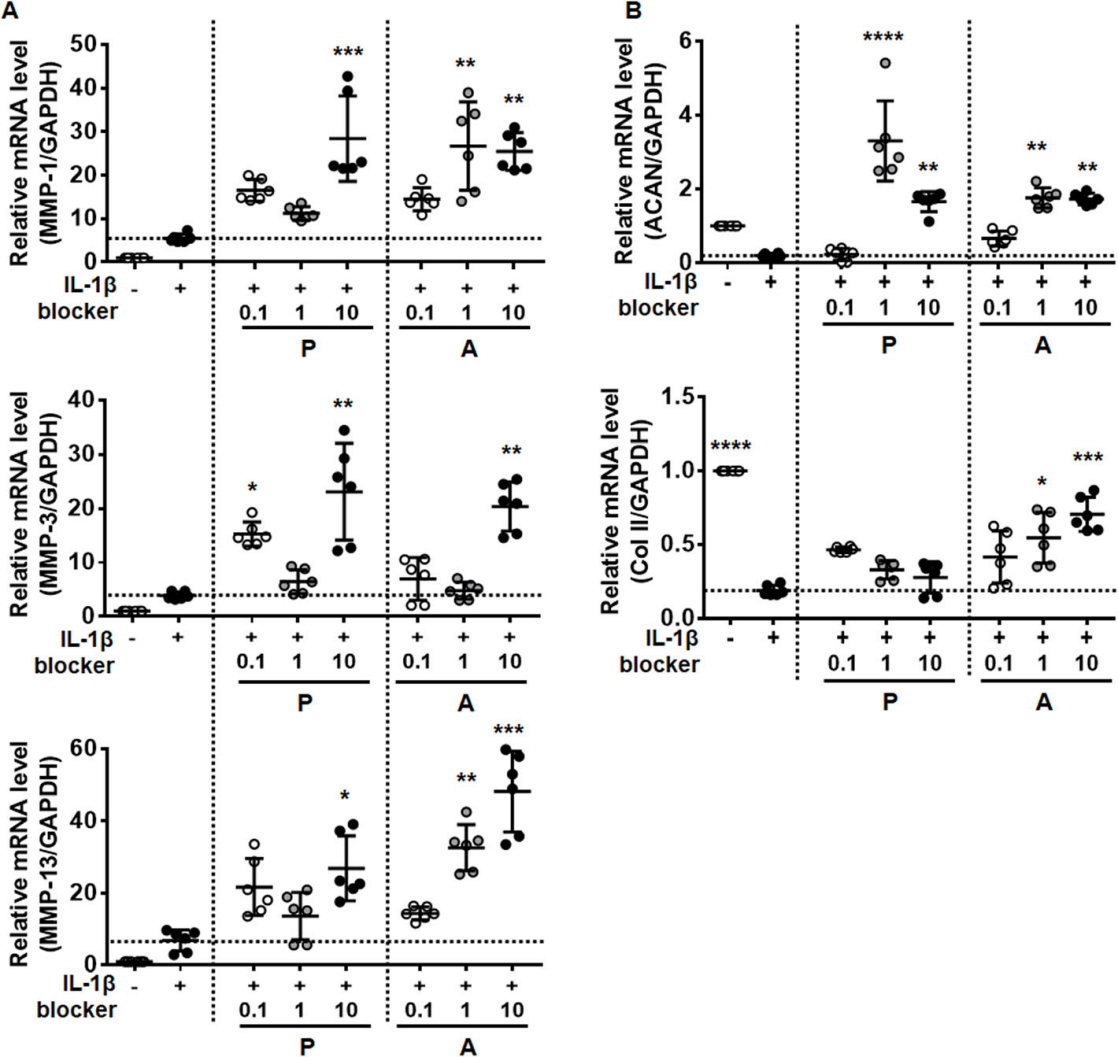
β-AR blockers affect IL-1β-induced expression of catabolic and anabolic factors. (**A**), (**B**) The effect of β-AR blockers on IL-1β-induced expression of (**A**) matrix degrading enzymes and (**B**) extracellular matrix proteins. Chondrocytes were pre-incubated with 0.1, 1.0, and 10 ng/ml of propranolol (P; non-selective β-adrenergic blocker) and atenolol (A; β1-adrenergic blocker) for 2 h followed by treatment with IL-1β (1 ng/ml) for 6 h. The mRNA levels of matrix degrading enzymes (MMP-1, -3, and − 13) and extracellular matrix proteins (ACAN and Col II) were measured using RT-PCR. Data represent the mean ± SD of duplicate data from three different donors. *P < 0.05, **P < 0.01, ***P < 0.001, and ****P < 0.0001 vs. IL-1β-treated cells by Kruskal-Wallis test with post hoc Dunn’s multiple comparison test

Sympathetic β-adrenergic blockers positively affect IL-1β-induced bone metabolism [12]. To first examine whether β-adrenergic blocker and agonist (NE) affect expressions of catabolic and anabolic factors, chondrocytes were incubated with β-adrenergic blocker and agonist (NE) alone for 48 h. β-adrenergic blockers and NE did not highly change MMPs expression but upregulated expression of anabolic factor aggrecan (ACAN) and collagen II (Col II) compared with untreated control cells (Fig. S2). To next test whether β-adrenergic blockers and NE modulate catabolic and anabolic factors expression in IL-1β-treated chondrocytes, chondrocytes were co-treated with IL-1β and β-adrenergic blockers (0.1, 1.0, and 10 µg/ml) for 6 h. Non-selective β-adrenergic blocker (propranolol) and β_1_-blocker (atenolol) used in our experiments significantly increased IL-1β-induced expression of MMP-1, -3, and − 13 especially at the highest concentration (Fig. 3A). In contrast, they reversed IL-1β-induced suppression of ACAN, while only atenolol had such influence on Col II (Fig. 3B). Contrary to 6 h treatment (Fig. 3), non-selective β-adrenergic blocker (propranolol and nadolol) and β1-blocker (atenolol and nebivolol) did not increase MMP expression compared to treatment with IL-1β alone after 48 h culture (Fig. S3A). β-adrenergic blockers did not affect ACAN and Col II regulation after 48 h of IL-1β treatment (Fig. S3B), suggesting that β-adrenergic blockers are effective only for the regulation of short-term response to IL-1β. Our data revealed that blockade of β-adrenergic receptors influenced chondrocyte response to IL-1β, such that it enhances both catabolic and anabolic markers.

**Fig. 4:**
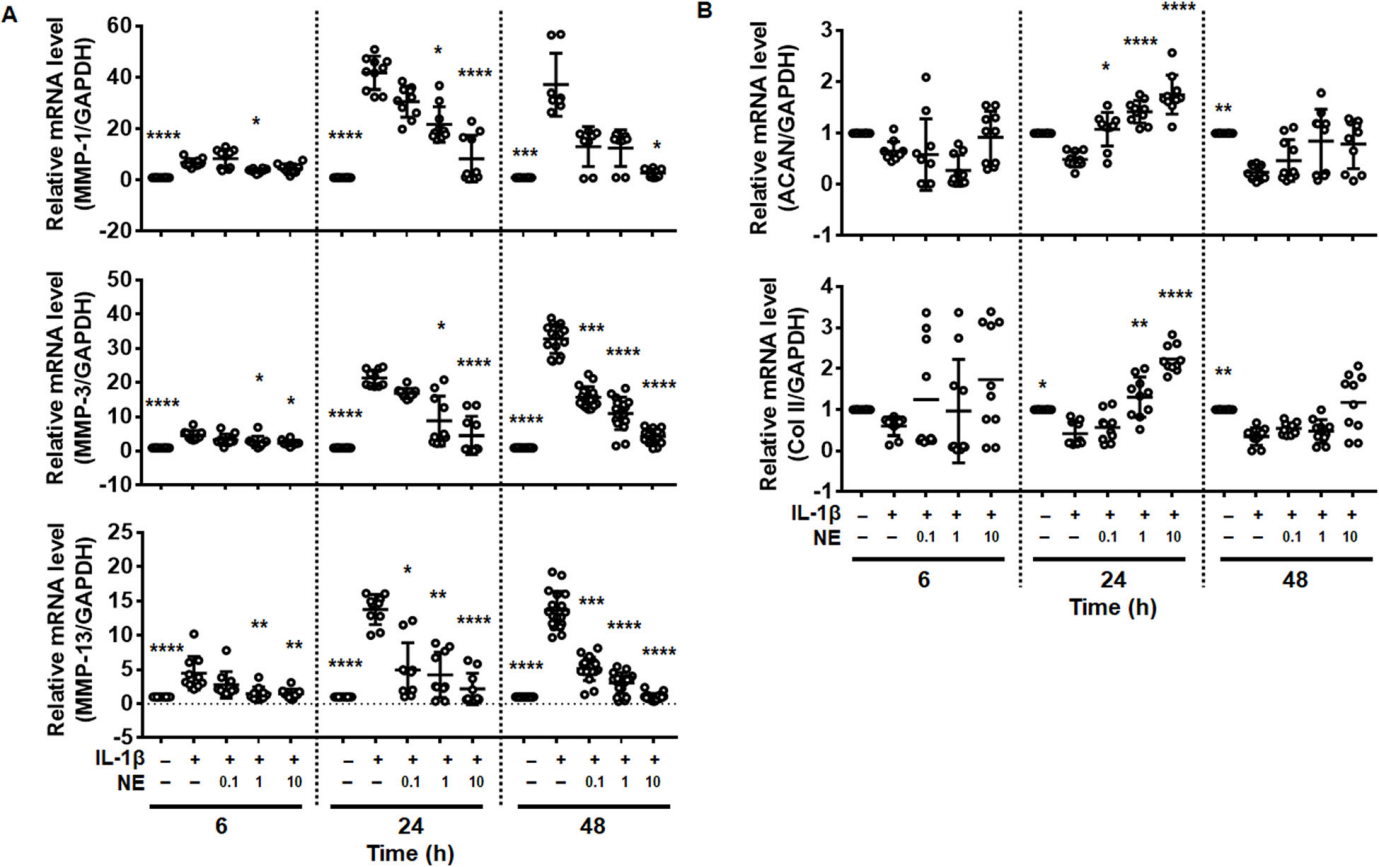
Norepinephrine (NE) reverses IL-1β-induced up-regulation of MMPs and down-regulation of matrix proteins. The effect of NE on IL-1β-induced expression of (**A**) MMPs and (**B**) extracellular matrix proteins. Chondrocytes were preincubated with NE (0.1, 1.0, and 10 ng/ml) for 2 h followed by treatment with IL-1β (1 ng/ml) for 6, 24, and 48 h. Data represent the mean ± SD of duplicate data from five different donors. *P < 0.05, **P < 0.01, ***P < 0.001, and ****P < 0.0001 vs. IL-1β-treated cells by Kruskal-Wallis test with post hoc Dunn’s multiple comparison test

**Fig. 5:**
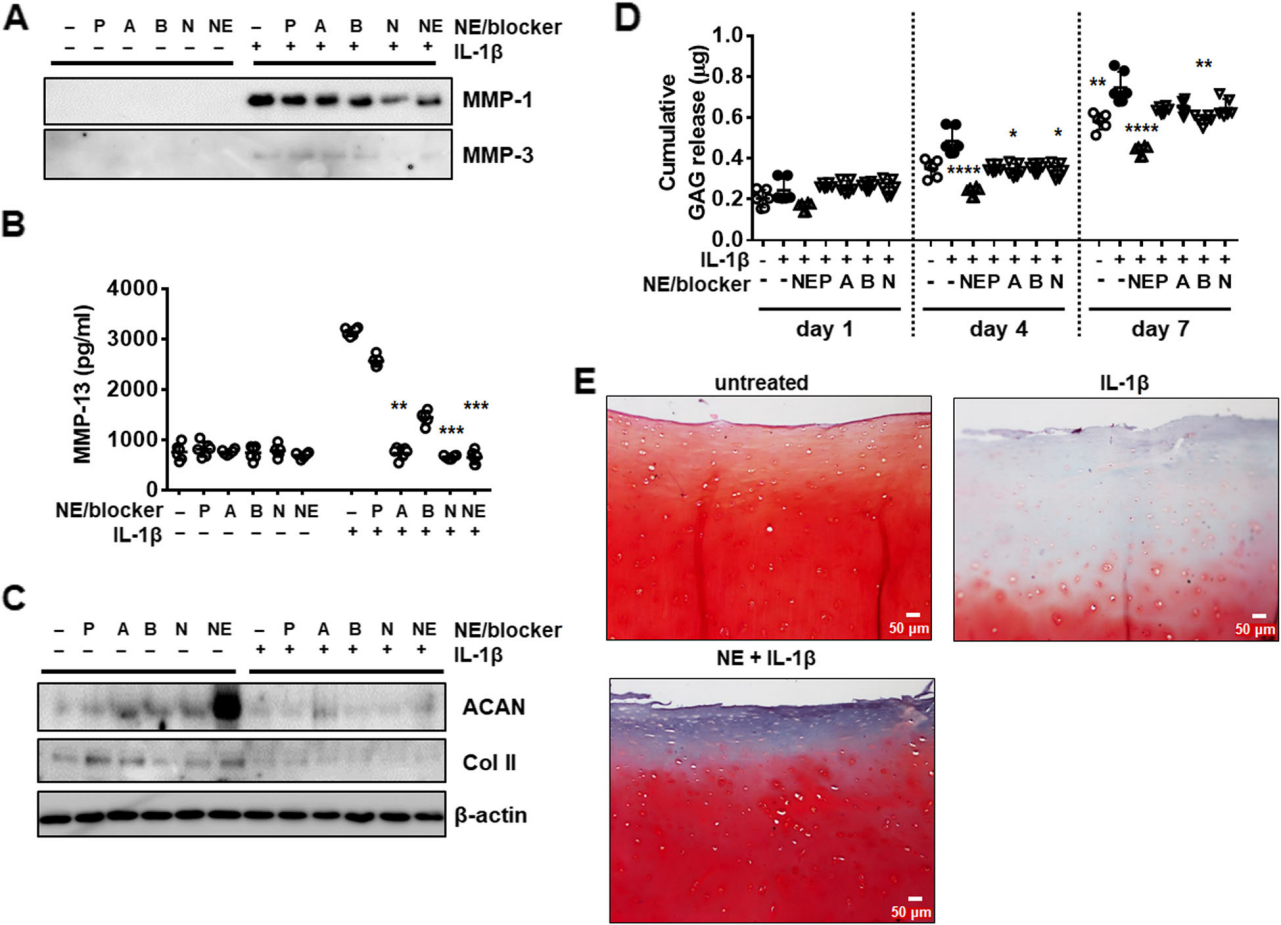
The effect of NE and β-AR blockers on IL-1β-induced expression of MMPs and extracellular matrix proteins. The effect of NE and β-AR blockers on IL-1β-induced expression of (**A**) MMP-1, -3, (**B**) MMP-13 and (**C**) extracellular matrix proteins (ACAN and Col II). Chondrocytes were pre-incubated with NE (1 ng/ml) or β-AR blockers (propranolol (P), atenolol (A), nebivolol (B), and nadolol (N); 1 ng/ml) for 2 h followed by treatment with IL-1β (1 ng/ml) for 48 h. Protein levels in the samples were measured using western blot analysis. β-actin was used as a loading control. (**D**), (**E**) NE suppresses IL-1β-induced release of glycosaminoglycan (GAG) into the medium in cartilage explant culture. (**D**) GAG release in cartilage explant culture treated with IL-1β and NE or β-AR blockers. Cartilage tissue (40 mg) was preincubated with NE (1.0 ng/ml) or β-AR blockers (P, A, B, and N; 1.0 ng/ml) 2 h before treatment with IL-1β (1 ng/ml) for 1, 4, and 7 days. NE or β-AR blockers were contained throughout the experiment. Culture medium was changed every two days for culture period. Concentration of GAG in culture medium was measured using Sulfated Glycosaminoglycan Assay kit. (**D**) Data represent the mean ± SD of duplicate data from three different donors. *P < 0.05, **P < 0.01, and ****P < 0.0001vs. IL-1β-treated cartilage tissue by Kruskal-Wallis test followed with post hoc Dunn’s multiple comparison test. (**E**) Safranin O staining of explant culture of human joint cartilage in the presence of IL-1β and NE. Scale bars = 50 μm

To next analyze the effect of NE on cartilage matrix metabolism, chondrocytes were treated with IL-1β with or without various concentrations (0.1, 1.0, and 10 µg/ml) of NE for 6, 24, and 48 h. IL-1β treatment increased MMPs expression in a time-dependent manner (Fig. 4A). Co-treatment with NE significantly inhibited IL-1β-induced expression of MMPs dose-dependently (Fig. 4A). NE led to reversal of IL-1β-induced down-regulation of ACAN and Col II after 24 h, which did not last after 48 h (Fig. 4B). The effect of β-adrenergic blockers or NE on protein levels of MMPs and ECM were measured in chondrocytes exposed to IL-1β for 48 h. The MMPs proteins were not induced in β-adrenergic blockers and NE alone (Fig. 5A-B), consistent with mRNA levels of MMPs (Fig. S2A). β-adrenergic blockers and NE alone significantly increased the level of ACAN protein compared with untreated control cell (Fig. 5C). Co-treatment with IL-1β and β-adrenergic blockers (A and N) or NE for 48 h reduced MMPs at the protein level compared with IL-1β-treated cells (Fig. 5A and B). These results demonstrate that activation of β-AR antagonizes IL-1β-induced catabolism of articular chondrocytes.

### NE suppresses IL-1β-induced release of GAG in cartilage explants culture

To investigate the effect of β-adrenergic agonist and blockers on ECM metabolism, GAG release was measured in medium from cartilage explants culture exposed to IL-1β with or without NE or β-adrenergic blockers for 1, 4 and 7 days. Untreated cartilage control showed time-dependent increase in cumulative GAG release (Fig. 5D). IL-1β treatment further increased GAG release into the medium compared with untreated control (Fig. 5D). NE significantly suppressed GAG release induced by IL-1β. GAG release in combinational treatment with IL-1β and β-adrenergic blockers (P, A, B, and N) for 4 and 7 days did not increase further compared to IL-1β-treated cartilage (Fig. 5D). In addition, we analyzed the level of ECM in cartilage tissue 7 days after explants culture using safranin O staining. IL-1β significantly decreased ECM level, whereas NE suppressed the reduction of ECM induced by IL-1β (Fig. 5E). These results demonstrate that activation of β-AR prevents loss of ECM in cartilage.

### NE suppresses IL-1β-mediated signaling pathways

**Fig. 6:**
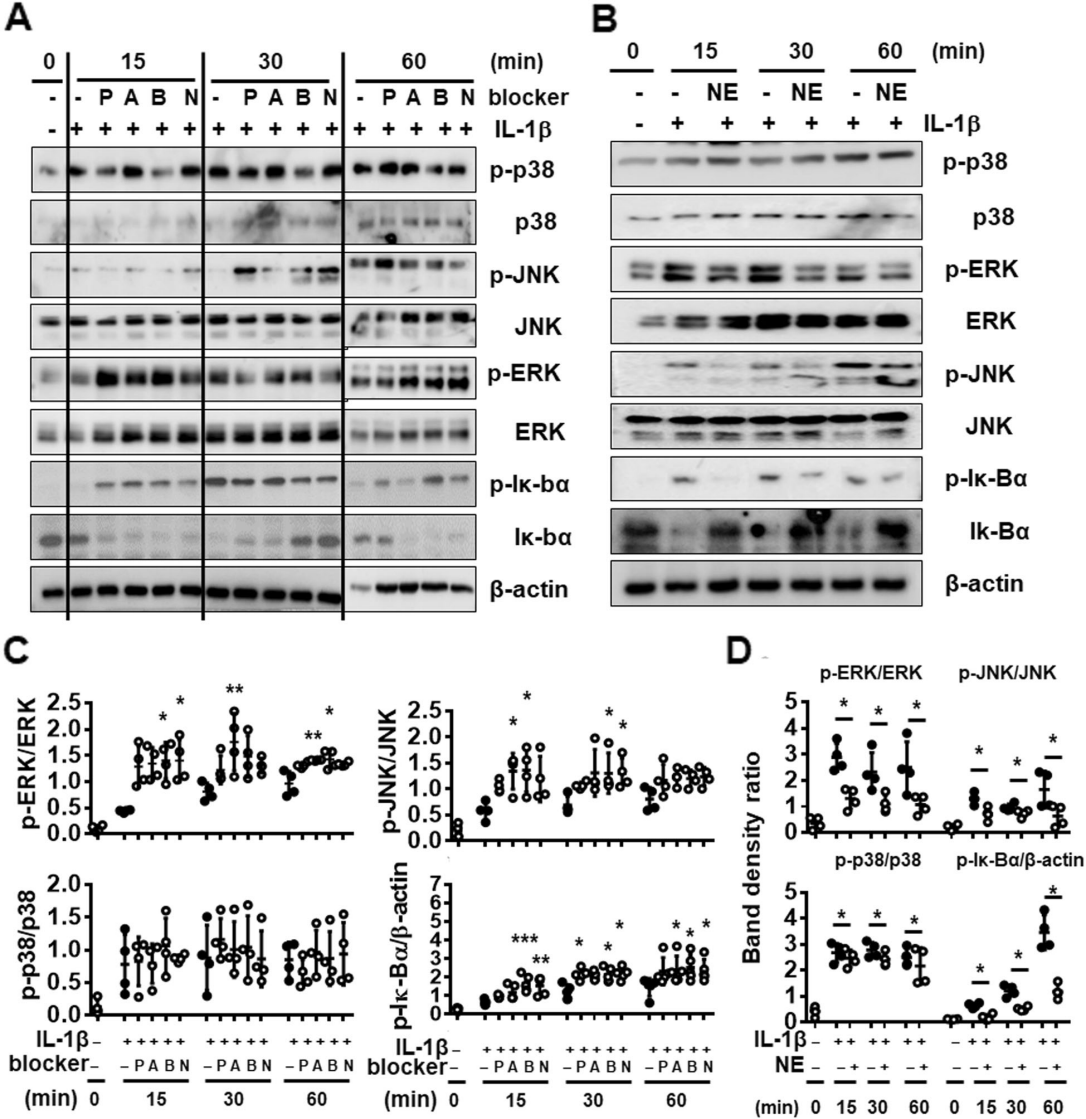
Effect of NE or β-AR blockers on IL-1β-activated signaling pathways in human chondrocytes. Chondrocytes were pretreated with (**A**) β-AR blockers (P, A, B, and N; 1.0 ng/ml) or (**B**) NE (1.0 ng/ml) 2 h before stimulation with IL-1β (1 ng/ml) for 15, 30, and 60 min. Phosphorylation of Jun N-terminal kinase (JNK), extracellular signal-regulated kinase (ERK), p38 MAPK, and Iκ-Bα was analyzed by Western blot. Western blots shown are representative of four independent experiments. β-actin was used as a loading control. Propranolol (P), atenolol (A), nebivolol (B), and nadolol (N). (**C**-**D**)The relative phosphorylation of JNK, ERK, p38, and Iκ-Bα. Protein band density was measured using Image J and normalized to the respective unphosphorylated protein, but β-actin in case of p-Iκ-Bα. Data represent the mean ± SD of data from four different donors. *P < 0.05, **P < 0.01, and ***P < 0.001 vs. IL-1β-treated cells by (**C**) Kruskal-Wallis test followed by Dunn’s multiple comparison test or (**D**) Mann-Whitney test

We investigated the effect of NE and β-adrenergic blockers on IL-1β-activated signaling pathways that contribute to MMPs, Col II, and ACAN expression. Chondrocytes were pre-incubated with NE and β-adrenergic blockers for 2 h and then treated with IL-1β for 15, 30, and 60 min. IL-1β induced phosphorylation of p38, JNK, and ERK MAPKs and Iκ-Bα (Fig. 6A and B). β-adrenergic blockers enhanced IL-1β-induced activation of JNK, ERK and Iκ-Bα (Fig. 6A and C), whereas NE significantly inhibited their activation by IL-1β (Fig. 6B and D). p38 activation was not affected. These results demonstrate that IL-1β-dependent activation of JNK and ERK MAPK and NF-κB is influenced by NE and β-adrenergic blockers in the opposite way.

## Discussion

β-ARs, target receptors of catecholamine involved in the SNS signaling, were down-regulated in both OA cartilage and IL-1β-treated chondrocytes compared with normal cartilage and untreated chondrocytes, respectively. In the present study we investigated the role of β-AR signaling in cartilage metabolism using β-adrenergic agonist and antagonists. Stimulation of β-AR facilitated overall anabolic responses by suppressing catabolic mediator expression and increasing cartilage matrix proteins in IL-1β-treated chondrocytes accompanied by down-regulation of ERK/JNK MAPK and NF-kB signaling pathway. On the contrary, blocking of β-AR led to short-term increase of IL-1β-mediated catabolic responses only. The decreased effect of β-blockers on IL-1β-induced GAG release in long-term treatment is likely attributed to alteration in signaling pathways associated with IL-1β and β-AR. These results show that SNS signaling via β-AR is closely related with regulation of cartilage metabolism.

The autonomic nervous system is composed of SNS and parasympathetic nervous system (PNS) that perform opposite actions. The SNS is responsible for the maintenance of homeostasis in response to harmful events leading to fight-or-flight responses [3]. During heart failure (HF), long-term increase in endogenous catecholamines, including NE and epinephrine, due to activation of the SNS plays a role in the progression of HF [14]. This is mediated via β-AR, and β-AR antagonists reduce morbidity and mortality in congestive HF. On the other hand, activation of β-ARs relaxes airway smooth muscle and inhaled β-AR agonists are considered as essential bronchodilator drugs in the treatment of bronchial asthma [15].

Several clinical and animal model studies have investigated the influence of the SNS on the severity of arthritis. In antigen-induced arthritis mouse model, chemical sympathectomy and pharmacological blockade of AR reduced arthritis severity such as joint inflammation and arthritis score [16]. Depletion of catecholamines by sympathectomy and elimination of sensory afferents by administration of capsaicin decreased joint injury in arthritic rats [17]. Patients with  rheumatoid arthritis exhibits milder pain by sympathetic blockade using guanethidine [18]. In murine chondrocyte monolayer culture, stimulation of β_2_-AR by isoproterenol inhibits Col X and Indian Hedgehog (Ihh) mRNA level through activation of ERK1/2 MAPK [19]. Sox-6 and Col II expression was inhibited by stimulation of β_2_-AR and this inhibitory effect was suppressed by propranolol, β_2_-AR antagonist [20], indicating that activation of β-AR signaling has negative effects on matrix protein synthesis in chondrocytes. By contrast, we found that β_1_-, β_2_-, and β_3_-AR expressions were reduced in OA cartilage relative to normal cartilage and pro-inflammatory cytokine IL-1β down-regulated three subtypes of β-AR compared to untreated control. In addition, our data showed that β-AR agonist prevented loss of ECM through decreased expression of pro-catabolic factors and increased expression of anabolic factors. These contradictory results are likely to be due to difference in types and concentrations of AR agonists and antagonists, AR subtypes interacting with them, and subsequent downstream signaling pathways.

In line with our results showing the suppression of IL-1β-induced catabolic responses by NE in monolayer and explant culture of human chondrocyte, several reports have demonstrated that catecholamines, including NE and dopamine, a precursor of NE, had anti-inflammatory effects in a variety of cells and *in vivo* models. NE reversed cartilage catabolism and inflammatory responses stimulated by IL-1β [9]. Adoptive transfer of tyrosine hydroxylase-positive neuronal cells generated from mesenchymal stem cells which exhibit a typical catecholaminergic phenotype led to markedly reduced severity of collagen induced arthritis in mice [21]. Dopamine prevented cartilage degradation in a DMM-induced OA mouse model and reduced MMPs expression and elevated Col II expression in IL-1β-treated chondrocytes via NF-κB and JAK2/STAT3 signaling pathway [22]. In lipopolysaccharide-stimulated microglia cells, dopamine suppressed nitric oxide production [23].

OA is a joint degenerative disease affecting entire joint tissue, including cartilage, synovium, subchondral bone, and menisci. The pathogenesis of OA was relevant to synovial inflammation, osteophyte formation, and subchondral bone sclerosis [24]. Because the subchondral bone marrow, the periosteum, synovium, and meniscus in joint are innervated compared to articular cartilage, many studies have mainly focus on role of the SNS and neurotransmitters in joint tissues, rather than cartilage tissue. Bone remodeling is also under sympathetic control, such that β-blocker treatment enhanced bone mass in wild type and ovariectomized mice [11]. Clenbuterol, a β_2_-AR agonist, suppressed longitudinal growth of bones in young rats together with muscular hypertrophy [25]. On the contrary, another study showed that clenbuterol relieved sciatic nerve injury-induced loss of bone mineralization [26]. Regulation of the SNS by leptin via β_2_-AR decreases osteoblast number and increases osteoclast differentiation leading to reduction of bone mass in mice [27, 28]. β_2_-AR-deficient mice exhibited greater bone mass in response to mechanical loading compared to wild type, but it was not shown in β_1_-knockout and β_1,2_-AR double knockout mice. In addition, administration of isoproterenol a non-selective β-AR agonist to wildtype mice increased bone resorption, indicating the possibility that β_1_- and β_2_-AR could play opposite role in bone metabolism [29]. It is of note that NE synthesizing enzymes and NE (at the concentration rage of 10^− 9^ to 10^− 7^ M) were detected in synovial tissue and fluids [4, 30–32], and β-ARs were expressed in the cartilage tissue and its surrounding tissues, including synovial macrophage and fibroblast, subchondral bone, and immune cells [33–35]. Based on others and our data demonstrating the presence of β-ARs in cartilage tissue and primary chondrocytes, the β-AR subtypes on joint tissues could be differently activated depending on the concentration of NE, indicating that β-AR-mediated signaling could contribute to the pathogenesis of OA.

It is also noted that the SNS is closely related with regulation of inflammatory responses, e.g. proinflammatory cytokine production. In a study of postmenopausal women with hypertension, central blockade of the SNS with moxonidine reduced serum TNF-α level [36]. TNF production is regulated through stimulation of α-AR or β-AR by catecholamines such as NE [37, 38].

NF-κB pathway is an inducible transcription factor involved in inflammation and cellular differentiation, and in ECM homeostasis [39]. In particular, the activation of NF-κB leads to increase in catabolic gene expression such as MMPs, ADAMTS5, and proinflammatory mediators, including cyclooxygenase-2 and inducible nitric oxide synthase. We found that regulation of β-AR activity with NE and four antagonists of AR had opposite influence on activation of JNK and ERK MAPK and NF-κB compared to IL-1β-treated chondrocytes, subsequently leading to alteration in expression of anabolic and catabolic factors. Thus, the SNS modulated pro-catabolic and anti-anabolic responses induced by IL-1β through regulation of JNK and ERK MAPK and NF-κB in chondrocytes. In addition, it is likely that β-ARs induce different biological responses through activating diverse downstream signaling molecules, including adenylate cyclase, depending on types, concentration, and treatment time of agonists and antagonists.

## Conclusions

In conclusion, our findings demonstrate that β-AR agonist NE converted IL-1β-driven catabolic responses to cartilage anabolism in articular chondrocytes. Cartilage homeostasis is likely to be closely related with the SNS level and activity. Whether modulators of the SNS signaling may be beneficial for prevention of OA progression should be studied in further animal experiments.

## Supplementary Information

**Additional file 1.**

## Data Availability

The datasets used and/or analysed during the current study available from the corresponding author on reasonable request.
