# Supplementary material for: Norepinephrine modulates IL-1β-induced catabolic response of human chondrocytes
Source: BMC Musculoskelet Disord. 2021 Aug 23;22:724. doi: 10.1186/s12891-021-04598-7 (PMC8383445; doi:10.1186/s12891-021-04598-7)
Supplement: Supplementary file 1 — Additional file 1. [file 12891_2021_4598_MOESM1_ESM.pdf]

Norepinephrine modulates IL-1β-induced catabolic response of human chondrocytes

Hyun Sook Hwang[a, b], Mi Hyun Lee[a,b], Dong Jin Go[c,*], Hyun Ah Kim[a,b,*]

[a] Division of Rheumatology, Department of Internal Medicine, Hallym University Sacred Heart Hospital, Kyunggi, 14068, Korea; [b] Institute for Skeletal Aging, Hallym University, Chunchon, 24251, Korea; [c] Division of Rheumatology, Department of Internal Medicine, Hallym University Kangnam Heart Hospital, Seoul, Korea,

Table S1. Primer sequences

Gene	Primer sequence
β_1-AR	Forward: 5’-TGC-TAC-AAC-GAC-CCC-AAG-TG-3’<br>Reverse: 5’-AGG-TAC-ACG-AAG-GCC-ATG-ATG-3’
β_2-AR	Forward: 5’-TTG-AAG-GCC-TAT-GGG-AAT-GG-3’<br>Reverse: 5’-TCC-ACT-CTG-CTC-CCC-TGT-GT-3’
β_3-AR	Forward: 5’-GCC-TTC-GCC-TCC-AAC-ATG-3’<br>Reverse: 5’-CAC-GAG-AAG-AGG-AAG-GTA-GAA-GGA-3’
ACAN	Forward: 5’-GGA-AGG-CTG-CTA-TGG-AGA-CAA-3’<br>Reverse: 5’-GGT-GTC-TCG-GAT-GCC-ATA-CG-3’
Col II	Forward: 5’-ACT-GGA-TTG-ACC-CCA-ACC-AA-3’<br>Reverse: 5’-TCC-ATG-TTG-CAG-AAA-ACC-TTC-A-3’
MMP-1	Forward: 5′-AGT-GAC-TGG-GAA-ACC-AGA-TGC-TGA-3′<br>Reverse: 5′-GCT-CTT-GGC-AAA-TCT-GGC-GTG-TAA-3′
MMP-3	Forward: 5′-GCG-TGG-ATG-CCG-CAT-ATG-AAG-TTA-3′<br>Reverse: 5′-AAA-CCT-AGG-GTG-TGG-ATG-CCT-CTT-3′
MMP-13	Forward: 5′-AAG-GAC-CCT-GGA-GCA-CTC-ATG-TTT-3′<br>Reverse: 5′-TGG-CAT-CAA-GGG-ATA-AGG-AAG-GGT-3′
GAPDH	Forward: 5′-TGA-TGA-CAT-CAA-GAA-GGT-GGT-GAA-G-3′<br>Reverse: 5′-TCC-TTG-GAG-GCC-ATG-TGG-GCC-AT-3′

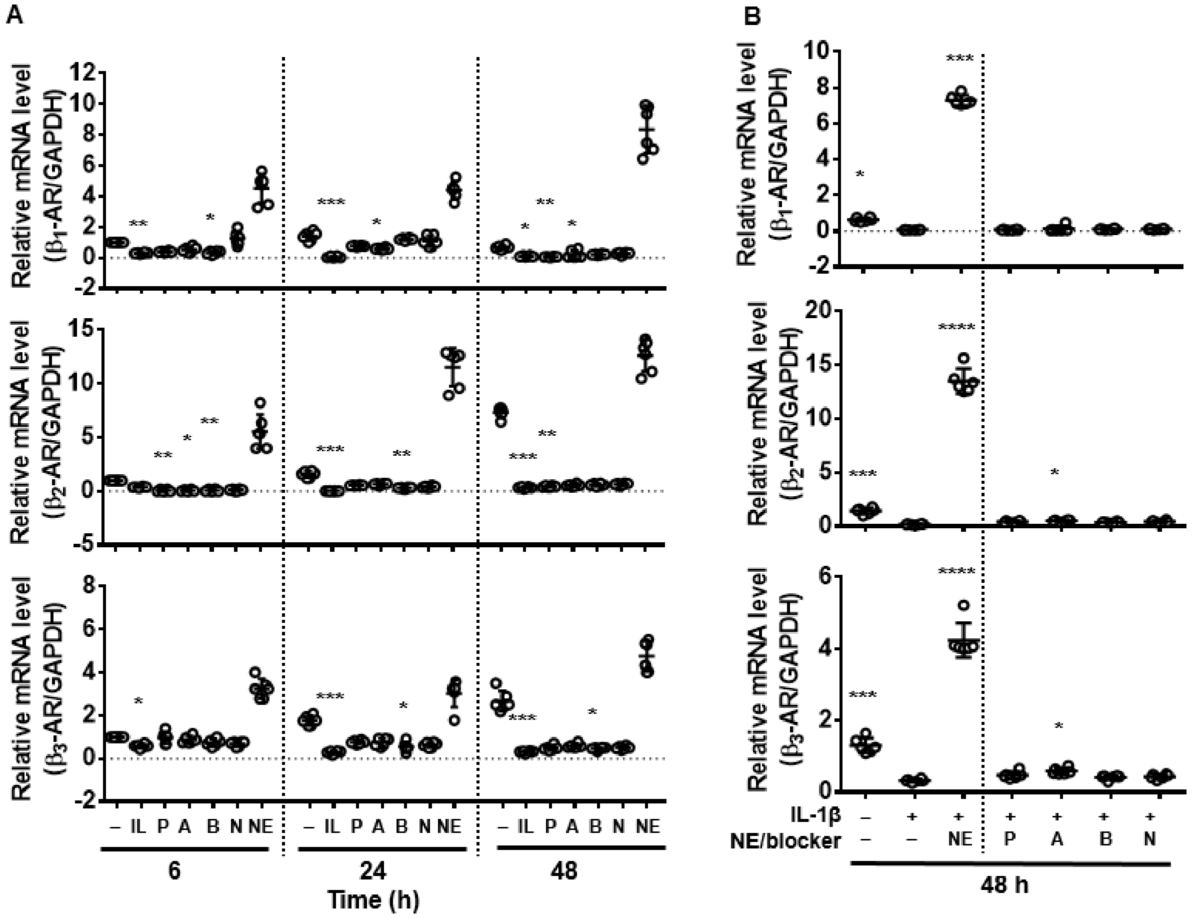

Figure S1. The effect of NE and β-AR blockers on mRNA expression of ARs with or without IL-1β. Chondrocytes were preincubated with NE (1 ng/ml) or β-AR blockers (propranolol (P), atenolol (A), nebivolol (B), and nadolol (N); 1 ng/ml) for 2 h followed by treatment with IL-1β (1 ng/ml) for 48 h. Data represent the mean ± SD of duplicate data from more than three different donors. **P* < 0.05, ***P* < 0.01, ****P* < 0.001, and *****P* < 0.0001 vs. (A) untreated and (B) IL-1β-treated cells by Kruskal-Wallis test with post hoc Dunn's multiple comparison test.

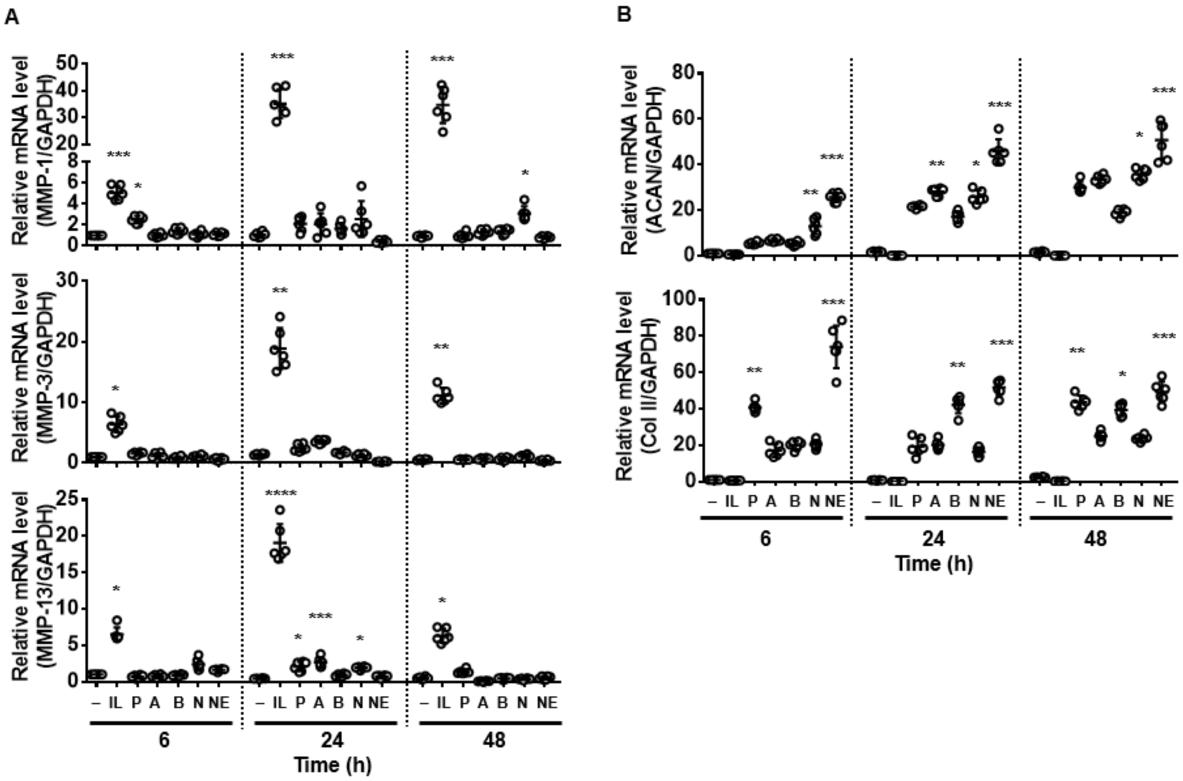

Figure S2. β-AR blockers and NE upregulate ECM with little influence on expression of matrix degrading enzymes. The mRNA expression of (A) matrix degrading enzymes (MMP-1, -3, and -13) and (B) extracellular matrix proteins (ACAN and Col II) in chondrocytes treated with β-AR blockers or NE alone. Chondrocytes were treated with 1 ng/ml of propranolol (P), atenolol (A), nebivolol (B), nadolol (N), and norepinephrine (NE) for 6, 24, and 48 h. The mRNA expression of matrix degrading enzymes (MMP-1, -3, and -13) and ECM proteins (ACAN and Col II) were measured using RT-PCR. Data represent the mean ± SD of duplicate data from more than three different donors. **P* < 0.05, ***P* < 0.01, ****P* < 0.001, and *****P* < 0.0001 vs. untreated control cells by Kruskal-Wallis test with post hoc Dunn's multiple comparison test.

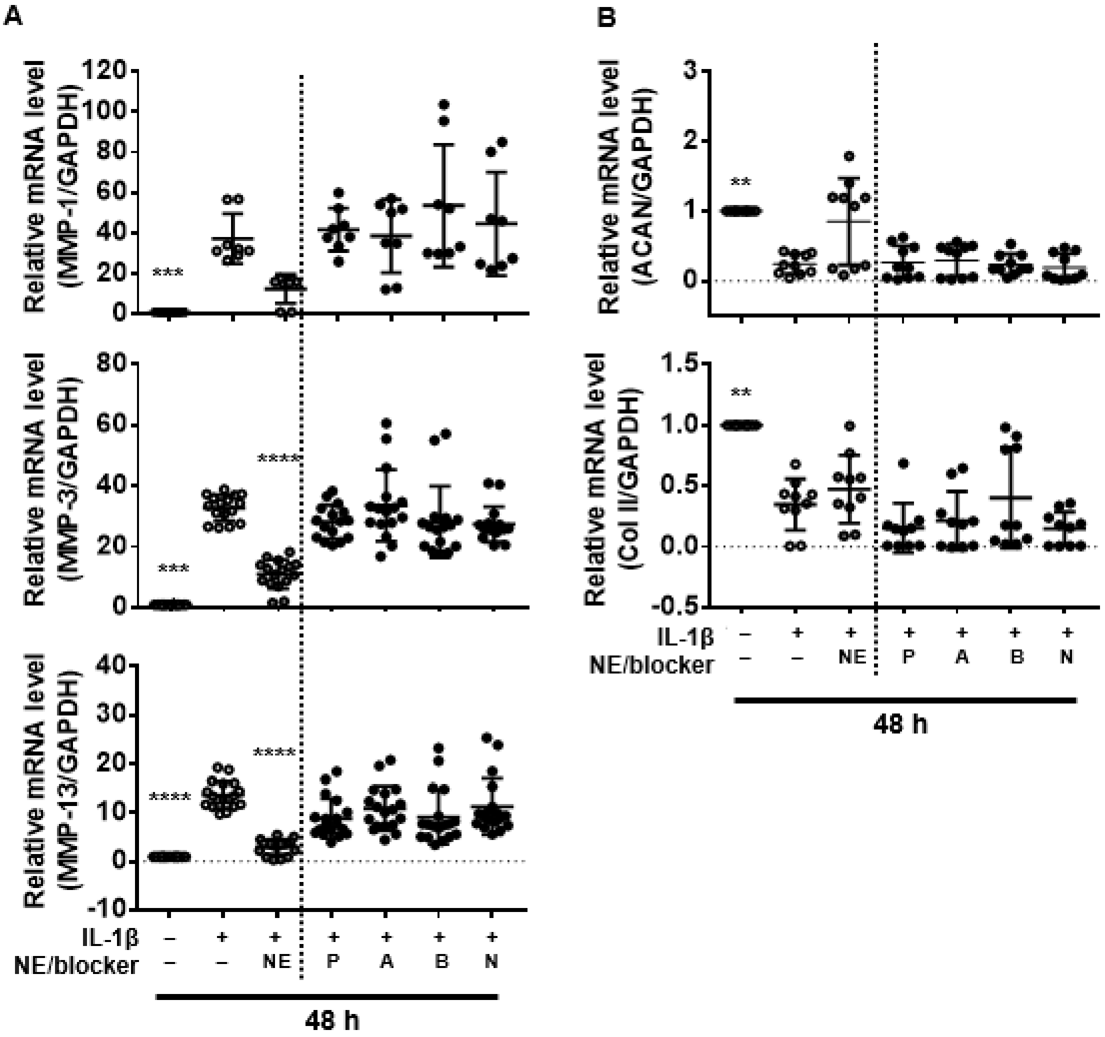

Figure S3. The effect of NE and β-AR blockers on mRNA expression of MMPs and extracellular matrix proteins in the presence of IL-1β. Expression of (A) MMPs and (B) ECM in chondrocytes co-treated with IL-1β and NE or β-AR blockers. Chondrocytes were preincubated with NE (1 ng/ml) or β-AR blockers (propranolol (P), atenolol (A), nebivolol (B), and nadolol (N); 1 ng/ml) for 2 h followed by treatment with IL-1β (1 ng/ml) for 48 h. Data represent the mean ± SD of duplicate data from more than three different donors. ***P* < 0.01, ****P* < 0.001, and *****P* < 0.0001 vs. IL-1β-treated cells by Kruskal-Wallis test with post hoc Dunn's multiple comparison test.

Original blots

Fig 2D

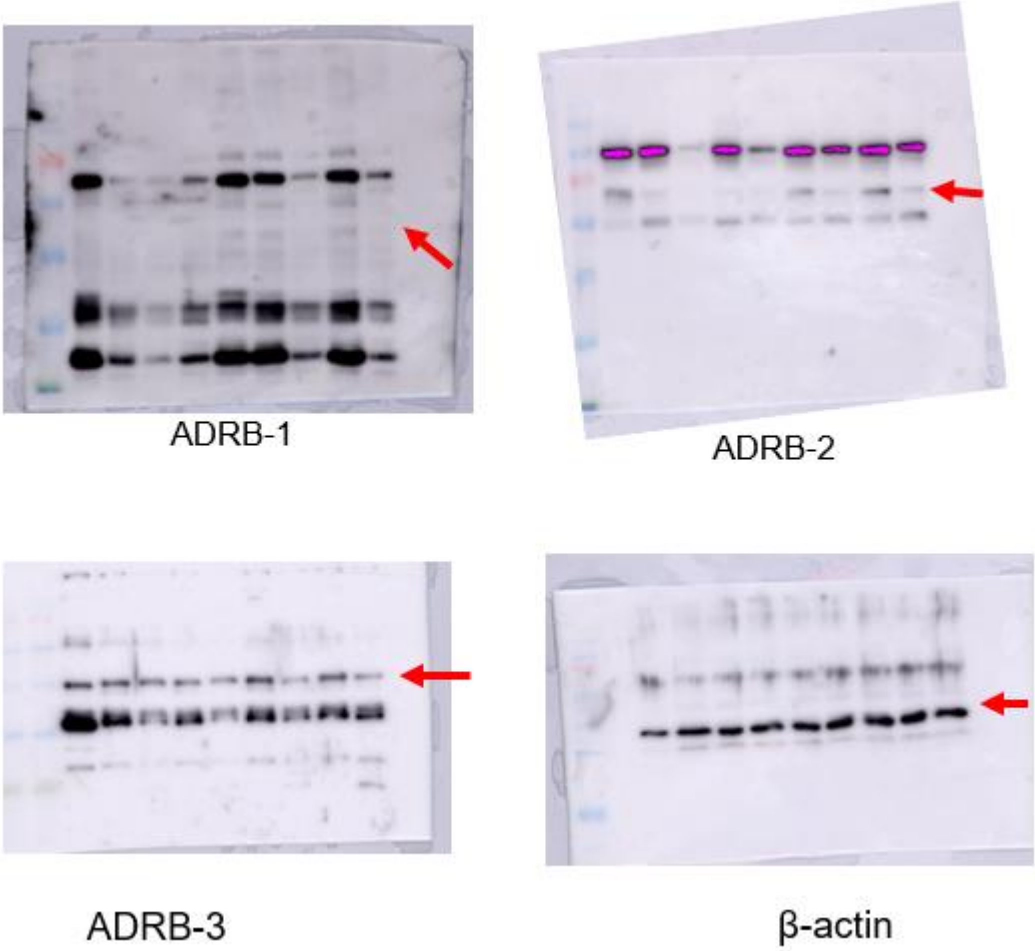

Figure 5A and C

Fig. 5A

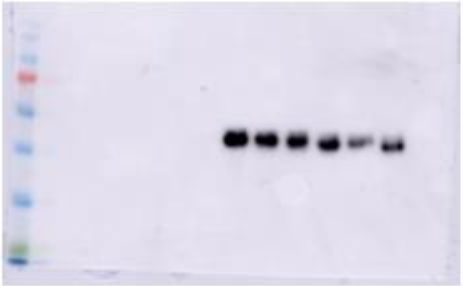

MMP-1

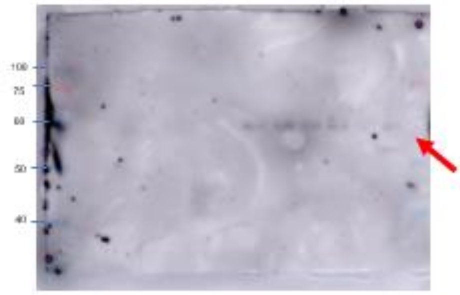

MMP-3

Fig. 5C

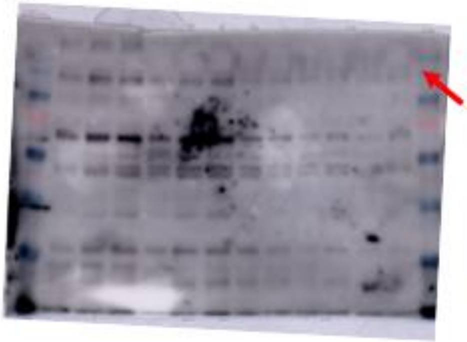

Col II

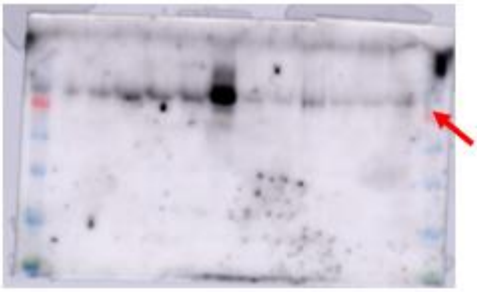

ACAN

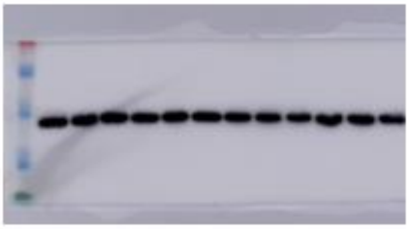

β-actin

Fig 6A

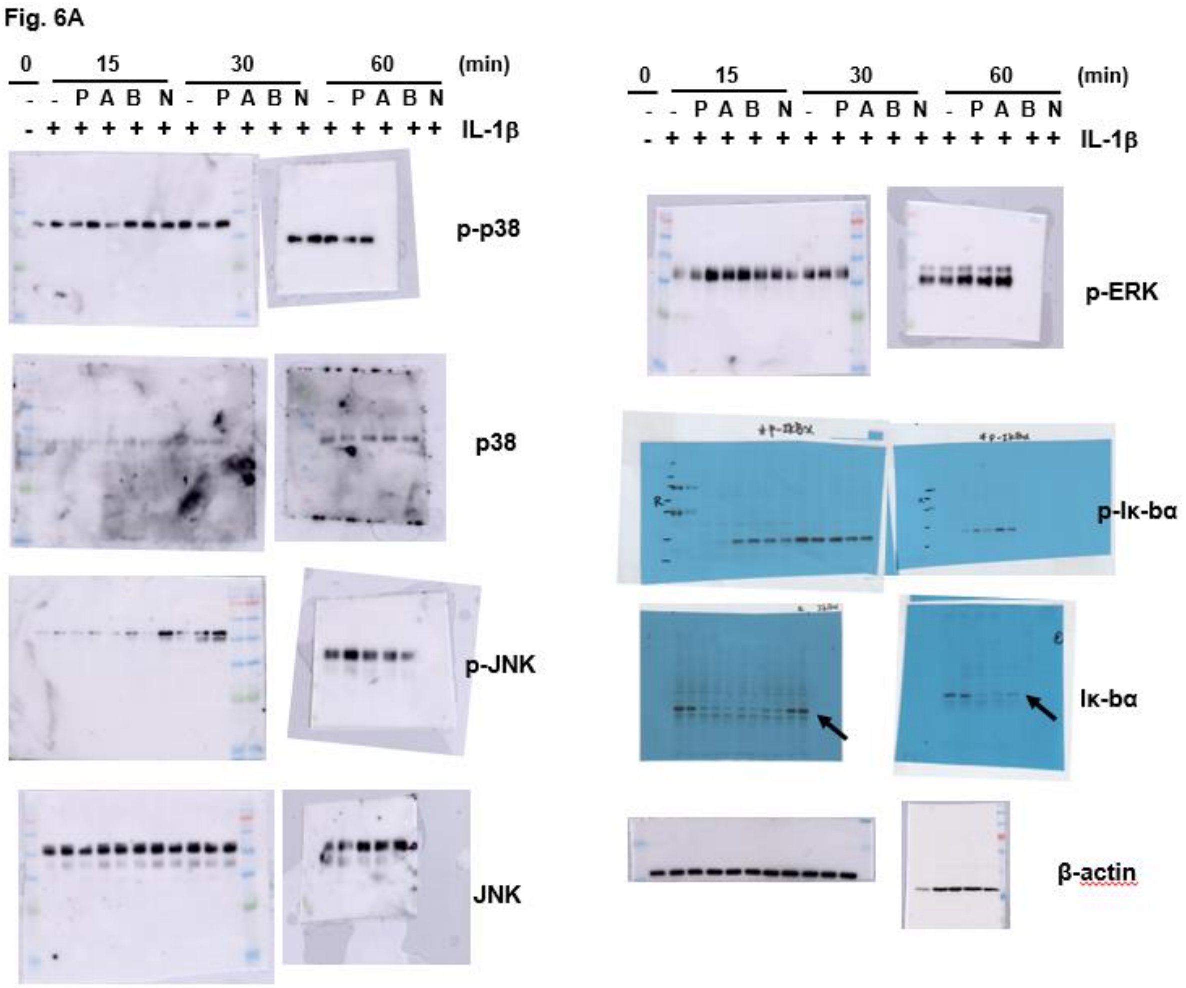
Fig. 6A
0
15
30
60
(min)
- - P A B N - P A B N - P A B N
- + + + + + + + + + + + + + + + +
IL-1β
p-p38
p38
p-JNK
JNK
p-ERK
p-Iκ-bα
Iκ-bα
β-actin

Fig 6B

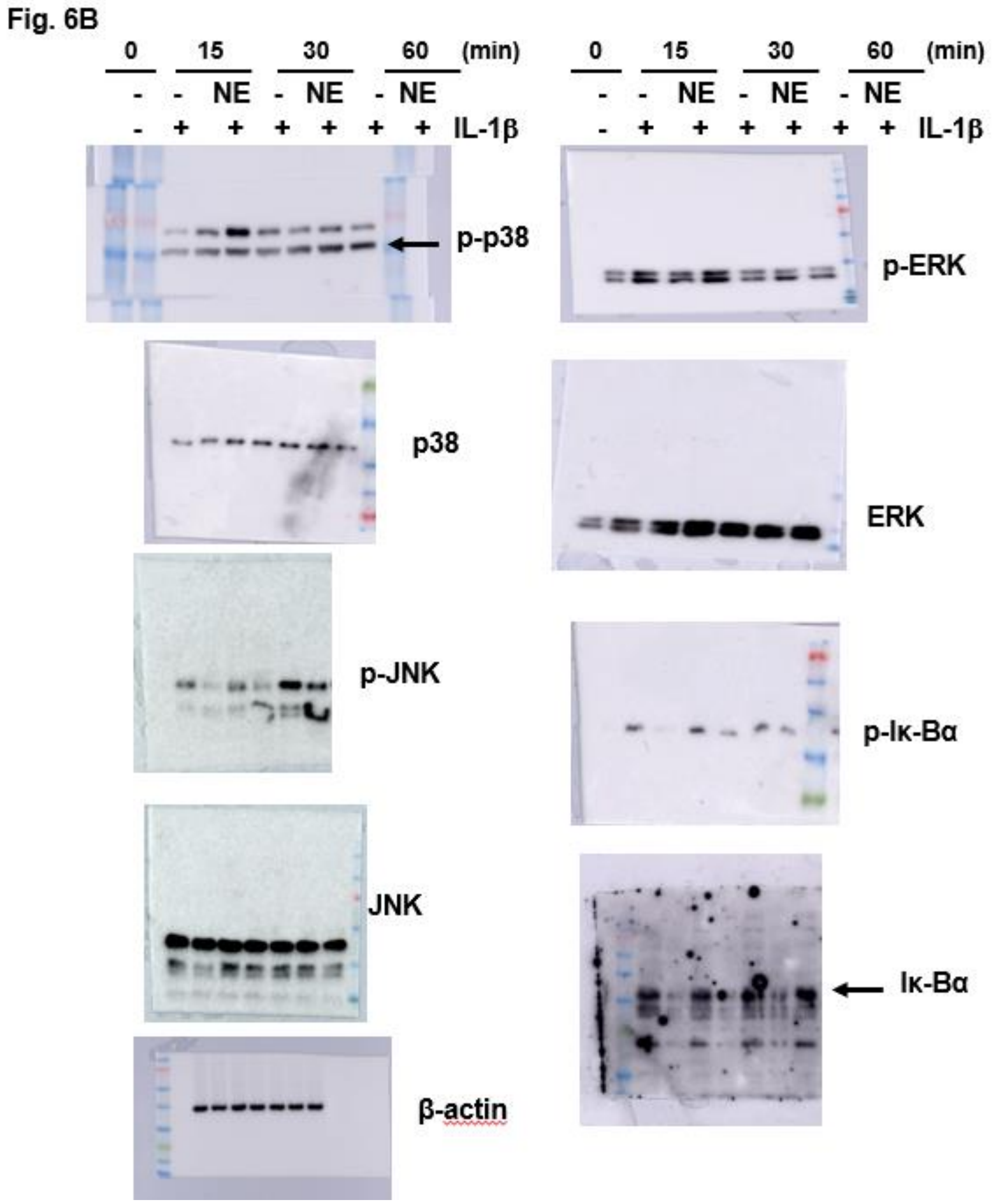
Fig. 6B
0
15
30
60
(min)
- - NE - NE - NE
- + + + + + + IL-1β
p-p38
p38
p-JNK
JNK
β-actin
0
15
30
60
(min)
- - NE - NE - NE
- + + + + + + IL-1β
p-ERK
ERK
p-Iκ-Bα
Iκ-Bα
